# The Book World of Medicine and Science

**Published:** 1897-02-20

**Authors:** 


### The Book World of Medicine and Science.

Charity Organisation and Jesus Christ. By Rev. C. L. Marson, M.A. (London: Scientific Press. Price 1s.)

Mr. Marson professes that he writes as "a bigot" to cast "anathemas" on scientific charity. From bigots useful guidance is hardly to be expected, but their writings have sometimes been interesting for the instance they show of sublime audacity or diabolic cleverness. Mr. Marson is a bigot, but his book is not interesting on account of its audacity nor of its cleverness. He is, indeed, audacious enough to fill up a charity organisation society's case-paper with an application from our Lord, but it is done with the triviality of a pedant and not with the passion of an enthusiast. His want of taste, or, rather, of sympathy, with common feelings has no compensation in the description of the applicant as a sort of socialist reformer, nor in the hard names—such as Miss Prudence Shockable—with which he loads members of the committee, nor in the decision which warns the applicant to adopt more business-like and practical habits. There is nothing in this which is sublime or clever. It is mere window breaking. The windows cover some of the tenderest human feelings, and the stones are those which any boy or monkey could pick from the road side. We ourselves do not profess to be uncritical admirers of the Charity Organisation Society. There is much in its methods which do not seem to represent Christian love; there is a stiffness about its so-called principles which tend to make the givers self-righteous and the receivers ungrateful; there is such a regard for "cases," "reports," that persons run the risk of being forgotten. Mr. Marson had, therefore, a fine opportunity when he set himself to compare the scientific charity of our day with that which flows from Jesus Christ. He sets about using his opportunity by quotations from writers of the early and middle centuries. He at once identifies Christianity with what he calls the Church, and what he calls the Church seems to have ceased to speak with authority when their writers ceased to write. It may well have been that in the days of St. Chrysostom "universal almsgiving" represented the spirit which was at war with the world of this day, but the same spirit teaches us to know various manifestations. As the world changes different acts become right. At one time a Christian master would have shown his Christian spirit by being kind to his slaves; at a later period he is forced by the same spirit to free his slaves. At one period of history the Christian lords rightly marched against the infidel, but at a later period they as rightly went as missionaries to the same people. That is to say, the spirit of love must work through the conditions made by new discoveries; just as the spirit of courage, which once showed itself in fierce hand-to-hand encounters, now shows itself in scientific warfare. The spirit of love raises life to new levels, and then has to adapt itself to what is done on those levels. It was kind to slaves, it did away with slaves, and now has to treat slaves as equals. It gave alms to every beggar; it has raised the beggar to the level of a brother, and it now has to treat him not with a penny or with a shilling, but with all the care, all the knowledge, which has been proved to be useful to a brother. The spirit of love, just as the spirit of courage, has to show itself in scientific charity. He would indeed give valuable guidance who would show us in detail how to put Christ's love into modern methods. We know that almsgiving is injurious. We know that the gifts of the monks drew idle crowds about the gates of the monasteries, and degraded men into loafers. We know that the Indian fashion, by which the industrious share their earnings with the idle, is one of the sources of famine; we know that Mansion House funds and out-relie demoralise whole neighbourhoods. It is no help to tell us that such methods are directed by the Church, or that they helped the friars to become holier and more loving. We might as well send out our armies clad in armour and equipped with bows and arrows, because great leaders of old times won battles by such means. Acts right in an age of ignorance are wrong in an age of knowledge. The question is how to put the spirit of utter giving—the spirit of Jesus Christ—into acts we know to be wise. We agree that this spirit is absent in much of the inquiry, organising, and good administration of which the present time boasts. We cannot go back to the old ways of indiscriminate giving. What are Christians to do? Mr. Marson does not tell us.

The Progress of Medical Chemistry. By J. L. W. Thudichum, M.D., F.R.C.P. (London: Baillière, Tindall, and Cox. 1896. Pp. 212. Price 5s.)

This is a book with a purpose. It is a vindication of Dr. Thudichum's long-cherished belief that he is the sole depository of the secrets of the great science of bio-chemistry. In his preface he attributes the decadence (discovered by him) of this science to the malevolent efforts of the late Professor Hoppe-Seyler, on whom he again pours the vials of his wrath in an indignant polemic extending over pages 28-34. But the famous Strasburg professor is by no means alone in the pillory; pages 99-104 are devoted to vituperation of Professor Salkowsky (who, in spite of this, is quoted with approval on page 20, when he happens to agree with the author), pages 157-9 to abuse of Kossel and Treytag, and page 193 to a most ungenerous attack on the late Professor Külz. Nor is English science exempt, for it is said of a research by the greatest physiological chemist this country has produced, Professor Gamgee, that "by this effort the blundering, so characteristic of modern physiology, reached a climax" (page 190). The scope of Dr. Thudichum's book is best indicated by quoting the title of Chapter IX., "On Supposed Carbohydrates as supposed sources of fatty acids from urine, and a new mode of obtaining an unearned increment of Literary Reputation by Mock Research"; or again of Chapter XIII., section 6, "How History is written by the Partisans of the Protagon Hypothesis." The only living physiological chemist quoted with approval (with, of course, the exception of the author himself) is Dr. Archibald Garrod, whose initials are incorrectly given on page 19. It would be ungenerous to criticise the grammar and spelling of a foreigner who has obviously not mastered the difficulties of our language; but it is at least legitimate to object to the substitution of new and often cacophonous names for those which have long been accepted, such as cruentin for hæmatoporphyrin, uropittine for urobibin, phrenosine for cerebrin, cohol for alcohol, &c., and to such "improvments" as, among others, molecle and baryum. We cannot withhold a final word of admiration for the publishers, who, as the preface states, have "generously taken the risk of the venture."

The Story of the Chemical Elements. M. M. Pattison Muir, M.A. 189 pages. (London: George Newnes. 1897.)

The value of this work would be more apparent if the author had informed us for what grade of student it is intended. The title is supplemented in the preface by the statement that "a few of the chief guiding conceptions of chemistry" are embodied in the book. The author tells us that his aim is to avoid technical details, and we find this one of the sources of our bewilderment. The book abounds in illustrations, which are references to or descriptions of chemical experiments. The beginner would, we imagine, find these of doubtful use, while the senior student, to whose notice we can nevertheless emphatically commend the book, would perhaps consider them a trifle long-drawn. In his desire to appeal to the scientific imagination of his readers, the author makes use of some singularly irritating illustrations. We doubt whether a mind to which they are necessary would be capable of grasping the conceptions to which they point.

The alchemist's views of the properties of matter are discussed at some length. We could wish they had been more shortly dealt with. Moral reflections occupy space which would have been better given to the subject in hand. For instance, "The study of chemistry humanises a man, and serves as a corrective to the brutalising tendencies of life, which are so numerous and so violent." It is, we suppose, possible to study chemistry in order to keep out of the public-house. It is disappointing to find a little book, which is in so many respects so excellent, in trivial ways going out of its way to be unattractive.

